# Association between skeletal muscle mass to visceral fat area ratio and arterial stiffness in Chinese patients with type 2 diabetes mellitus

**DOI:** 10.1186/s12872-018-0827-z

**Published:** 2018-05-08

**Authors:** Jing Xu, Xiaoyan Pan, Haili Liang, Yi Lin, Yilian Hong, Qiya Si, Feixia Shen, Xuejiang Gu

**Affiliations:** Department of Endocrine and Metabolic Diseases, 1st Affiliated Hospital of Wenzhou Medical University, Ouhai District, Wenzhou, Zhejiang Province 325000 People’s Republic of China

**Keywords:** Skeletal muscle mass, Visceral fat area, Skeletal muscle mass to visceral fat area ratio, Arterial stiffness, Type 2 diabetes mellitus

## Abstract

**Background:**

The skeletal muscle mass-to-visceral fat area ratio (SVR) has been linked to arterial stiffness in non-diabetic adults. We examined the association between the SVR and arterial stiffness in patients with type 2 diabetes mellitus (T2DM).

**Methods:**

Patients with type 2 diabetes mellitus (252 men and 171 women) aged 40–75 years were enrolled and divided into three groups according to SVR tertiles. Arterial stiffness was measured as brachial-ankle pulse wave velocity (baPWV), with baPWV> 1800 mm/s defined as high. Spearman’s partial correlation was used to adjust confounding factors. The odds ratio for high baPWV was determined by multiple logistic regression analyses, and receiver-operating characteristic analysis was conducted.

**Results:**

SVR was associated with baPWV in Chinese patients with T2DM (Spearman’s partial correlation = − 0.129, *P* < 0.01). SVR was found to be significantly associated with baPWV on multiple logistic regression analysis. Patients in the lower SVR tertiles had a higher OR than did those in the higher SVR tertiles, after adjusting for multiple covariates (Q1: OR = 4.33 in men and 4.66 in women; Q3: OR = 1). The area under the curve for SVR was significantly greater than that for appendicular skeletal muscle (ASM), ASM/height^2^, and visceral fat area (VAF) for identifying high baPWV (0.747 in men and 0.710 in women). The optimal cutoffs values of SVR for detecting high baPWV were 191.7 g/cm^2^ for men and 157.3 g/cm^2^ for women.

**Conclusions:**

SVR has an independent, negative association with arterial stiffness, and is a better risk-assessment tool than ASM, ASM/height^2^, and VFA in clinical practice to identify patients with type 2 diabetes at high cardiovascular risk.

## Background

For patients with type 2 diabetes, the major cause of morbidity and mortality is cardiovascular disease (CVD) [1]. As the traditional risk factors, including low high-density lipoprotein cholesterol (HDL-C) levels, high low-density lipoprotein cholesterol (LDL-C) levels, smoking, as well as hypertension fail to explain the increased cardiovascular risk in patients with T2DM completely [1], it is essential to develop a risk factor assessment tool that is clinically applicable, economical, and non-invasive.

Sarcopenia is associated with the atherosclerotic process and it has been proved that the levels of skeletal muscle mass of patients with type 2 diabetes present is lower than patients with no diabetes [2, 3]. On the other hand, as is well-known, visceral fat accumulation can increase the risk for CVD [4]. The phenomenon of sarcopenic obesity has been found recently, which indicates that sarcopenia and visceral obesity often coexists with each other. Previous studies have shown that sarcopenic obesity presents a dual metabolic burden and is emerging as a major concern for public health [5, 6]. However, most of previous studies have explored the role of sarcopenia and obesity separately; their synergistic effects on cardiovascular disease in T2DM patients are not clear. Therefore, here, we have evaluated and presented an index of sarcopenic obesity using the ratio of appendicular skeletal muscle (ASM) to visceral fat area (VFA), known as the skeletal-to-visceral ratio (SVR).

Arterial stiffness measured as brachial-ankle pulse wave velocity (baPWV) is an indicator of atherosclerosis and an independent predictor risk factor for all-cause mortality and cardiovascular mortality [7]. Some clinical studies have demonstrated an independent association between arterial stiffness and atherosclerosis [8].

The aim of this study was to explore the association between SVR, an index of skeletal muscle mass corrected by visceral fat area, and arterial stiffness in T2DM patients. We further evaluated the association of SVR, ASM, ASM/height^2^, and VFA with arterial stiffness, in middle aged and old patients with T2DM.

## Methods

### Subjects

The subjects in this cross-sectional study were 423 Chinese patients with T2DM (40 years≤ age ≤ 75 years). We consecutively enrolled the subjects who visited the first hospital affiliated with Wenzhou medical university for evaluation or treatment of T2DM. Patients with renal dysfunction (estimated glomerular filtration rate < 30 ml/min/1.73 m^2^), nutritionally compromise (such as cancer, thyroid disease, skeletal deformities and amputations), chronic heart failure, and atrial fibrillation were excluded from this study. Patients with peripheral artery disease whose ankle-brachial index< 0.9 were also excluded [9]. We have received the agreement from all subjects that they were willing to participate in this study and the informed consent was provided too. This study was approved by the Institutional Review Board of the first affiliated hospital of Wenzhou Medical University.

### Anthropometric and biochemical measurements

Physical examination included anthropometric measures (body height, weight, and waist circumference) and blood pressure assessment. The body mass index (BMI) was then calculated as weight (kg) divided by height squared (m^2^). Blood pressure (BP) (mmHg) was measured after a 5 min rest in the supine position, using a mercury sphygmomanometer. Glycosylated hemoglobin (HbA1c), total cholesterol (TC), triglyceride (TG), HDL-C, LDL-C, albumin, uric acid, fasting serum insulin (FINS), were measured after a fasting period of at least 8 h, using the auto analyzer.

### The application of a dual bioelectrical impedance analyzer to measure muscle mass and fat mass

The lean body mass of the arms and legs, ASM, total body fat percentage (BF), as well as VFA were measured with a dual bioelectrical impedance analyzer (InBody 720; Biospace, Seoul, Korea). Although VFA measured by a dual bioelectrical impedance analyzer is different from that measured by an abdominal computed tomography (CT), a good correlation between them has been demonstrated in the previous studies [10]. ASM (kg) was defined as the sum of the lean soft tissue masses of the arms and legs, according to Heymsfield et al. [11]. As it was described above, we also calculated the ASM adjusted by stature index (ASM/height^2^) [12]. By dividing the ASM (g) by VFA (cm^2^), SVR (g/cm^2^) was also calculated to be an index of sarcopenic obesity.

### Measurement of baPWV

In this study, we measured the baPWV as well as the brachial and ankle blood pressures on the left and right sides of the subjects with a plethysmography apparatus (model BP-203RPE II; Colin, Komaki, Japan) after rested in a supine position for 5 min. In this study, baPWV was calculated as the mean of the left and right baPWV values. High baPWV was defined as baPWV > 1800 mm/s [13].

### Statistical analysis

The T2DM patients were divided into sex-specific SVR tertiles (Q) as follows: Q1, < 182 g/cm^2^; Q2, 182 g/cm2–237 g/cm^2^; Q3, > 237 g/cm^2^ in men; and Q1, < 126 g/cm^2^; Q2, 126 g/cm^2^–156 g/cm^2^; Q3, > 156 g/cm^2^ in women. Numerical data are expressed as mean ± SD or median [inter-quartile range]. Categorical variables are presented as percentage. Comparisons of the means and proportions were performed by one-way ANOVA analysis and Pearson’s χ2-test, respectively. In this study, we adopted the partial correlation adjusting for age and gender of Spearman to estimate the correlation between SVR, ASM, ASM/height^2^, VFA, and other variables. Multiple logistic regression analysis was used to estimate significant trends across decreasing tertiles based on SVR and to estimate the odds ratio of high baPWV in each tertile compared with the highest tertile as the reference category. The regression models were adjusted for age, duration of diabetes, duration of hypertension, BMI, waist circumference, systolic blood pressure (SBP), diastolic blood pressure (DBP), HbA1c, smoking status, alcohol intake, FINS, uric acid, TC, TG, HDL-C, LDL-C. To evaluate the clinical utility of SVR in predicting arterial stiffness, the receiver operating characteristic (ROC) curve was plotted and the area under the curve was calculated.

All analyses were carried out using statistical computer programs, Med Calc version 12.3.0 (MedCalc Software, Broek-straat 52, 9030 Mariakerke, Belgium) and IBM SPSS version 20.0 (SPSS Inc., USA). A *p* value < 0.05 was considered significant.

## Results

### Comparisons of the general characteristics among T2DM patients with different tertiles of SVR

Clinical data and baseline characteristics of patients with different tertiles of SVR are presented in Table 1. Among both men and women, patients in the lower tertiles of SVR had a higher age, duration of hypertension, BMI, waist circumference, total body fat percentage, visceral fat area, and baPWV than did patients in the higher tertiles of SVR, and the significant differences were mainly between Q1 and Q2, and Q1 and Q3. In addition, the TC levels were significantly higher in women Q3 than in those in Q1. The TG, HDL, LDL, albumin, uric acid, and HbA1c levels were not significantly different among different SVR tertiles.

**Table 1:** Clinical, anthropometric and metabolic characteristics of patients according to SVR tertiles

	Men			women		
	Q1	Q2	Q3	Q1	Q2	Q3
N	82	86	84	56	57	58
Age, years	63.2±9.1	58.6±9.0^*^	51.7±7.6^*^	64.6±8.3	62.8±7.6	54.7±8.4^§^
Duration of diabetes, years	9.0±6.7	9.4±7.4	8.2±6.1	11.2±6.8	9.7±5.9	9.3±5.8
Duration of hypertension, years	5.5±6.8	3.8±6.4	2.1±5.6^*^	8.0±8.0	6.2±7.1	4.1±5.2^§^
Height, cm	165±6	169±5^*^	170±5^*^	154±5	155±5	157±6^§^
Weight, Kg	69±11	69±9	65±9^*^	63±11	59±9^§^	58±9^§^
BMI, Kg/m2	25.3±3.6	24.3±3.0^*^	22.7±3.0^*^	26.7±3.9	24.4±3.3	23.6±3.3^§^
Waist, cm	92.6±8.7	88.9±8.1^*^	84.8±8.2^*^	94.3±9.6	88.5±12.8^§^	82.3±8.4^§^
SBP, mmHg	143±22	136±19^*^	133±21^*^	143±22	145±26	144±24
DBP, mmHg	80±13	77±12	78±12	74±11	78±14	78±12^§^
TC, mmol/l	4.3±1.1.	4.3±1.2	4.3±1.2	4.6±1.4	4.6±1.2	5.1±1.5^§^
TG, mmol/l	1.6±0.8	1.8±1.3	1.8±2.1	1.9±0.9	2.0±1.2	2.5±3.3
HDL, mmol/l	0.9±0.2	0.9±0.2	1.0±0.9	1.0±0.2	1.0±0.2	1.1±0.3
LDL, mmol/l	2.5±0.9	2.5±0.9	2.4±0.9	2.6±1.1	2.5±0.8	2.9±1.0
Albumin, g	38.3±3.9	39.3±3.6	38.2±4.9	37.4±3.4	37.5±4.0	37.2±5.2
Uric, umol/l	335±95	339±107	322±87	309±85	286±86	299±92
HbA1c, mmol/l	10.1±2.2	9.5±2.4	10.5±3.0	9.2±1.9	9.2±1.9	9.9±2.2
FINS, pmol/l	139±330	60±65	40±37^*^	114±189	74±77	60±72
Smoking, %	52.4	36.0^*^	59.5	0	0	3.4
Alcohol intake, %	39.0	38.4	36.9	1.8	0	3.4
VFA, cm2	131±18	108±13^*^	78±17^*^	135±18	111±15^§^	84±18^§^
BF (%)	27.2±5.5	22.0±4.5^*^	16.4±4.5^*^	38.0±8.1	31.7±5.2	26.7±6.7^§^
ASM, Kg	20.4±2.7	22.4±2.3^*^	22.8±2.7^*^	14.7±2.7	15.3±2.0	16.3±2.4^§^
ASM/Height2, Kg/m2	7.4±0.7	7.8±0.7^*^	7.9±0.7^*^	6.8±4.3	6.3±0.8	6.6±0.8
SVR, g/cm2	157±17	209±16^*^	323±84^*^	109±16	138±57^§^	201±45^§^
baPWV	1813±441	1617±313^*^	1480±338^*^	2073±448	1852±281^§^	1740±407^§^

*Abbreviations*: *BMI* body mass index, *SBP* systolic blood pressure, *DBP* diastolic blood pressure, *TC* total cholesterol, *TG* triglyceride, *HDL-C* high-density lipoprotein cholesterol, *LDL-C* low-density lipoprotein cholesterol, *HbA1c* glycated hemoglobin A1c, *FINS* fasting serum insulin, *HOMA-IR* homeostatic model assessment of insulin resistance, *BF* total body fat percentage, *ASM* appendicular skeletal muscle, *VFA* visceral fat area, *SVR* skeletal muscle mass to visceral fat area ratio, *baPWV* brachial-ankle pulse wave velocity

^*^P < 0.05, vs Q1 in men group, ^§^*P* < 0.05, vs Q1 in women group

### Spearman’s partial correlations between body composition and other variables after adjustment for age and gender

**Fig. 1:**
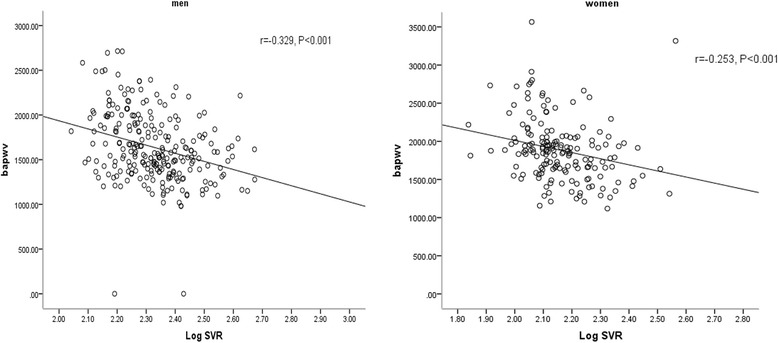
Relationship between SVR and mean brachial-ankle pulse wave velocity (baPWV). Men, *r* = − 0.329, *P* < 0.001. women, *r* = − 0.253, *P* < 0.001

Spearman’s partial correlations adjusted for age and gender are shown in Table 2. SVR had a negative association with BMI, waist circumference, DBP, SBP, baPWV (Fig. 1). ASM was significantly and positively correlated with BMI, waist circumference, TG, uric acid and negatively with HDL-C, HbA1c, baPWV. ASM/height^2^ was significantly and positively correlated with waist circumference and uric acid level. VFA was significantly and positively correlated with BMI, waist circumference, DBP, uric acid level, and baPWV and negatively correlated with HDL-C, HbA1c (all *P* < 0.05).

**Table 2:** age and gender adjusted spearman’s correlations between SVR, ASM, ASM/height2 and VFA and other variables

	SVR adjusted for age and gender		ASM adjusted for age and gender		ASM/height2 adjusted for age and gender		VFA adjusted for age and gender	
	r	P	r	P	r	P	r	p
BMI	−0.169	0.003	0.534	0.000	−0.047	0.412	0.687	0.000
Waist	−0.119	0.036	0.414	0.000	0.253	0.000	0.565	0.000
SBP	−0.071	0.216	0.057	0.315	0.016	0.784	0.105	0.066
DBP	−0.132	0.021	0.058	0.306	0.015	0.800	0.154	0.007
TC	−0.076	0.185	0.02	0.723	−0.043	0.452	0.015	0.789
TG	0.001	0.989	0.159	0.005	0.065	0.257	0.016	0.778
HDL-C	−0.005	0.925	−0.120	0.035	−0.106	0.064	−0.134	0.019
LDL-C	−0.087	0.126	−0.047	0.411	−0.063	0.270	0.033	0.567
Uric	0.021	0.710	0.147	0.01	0.180	0.002	0.161	0.005
HbA1c	0.047	0.411	−0.121	0.033	−0.078	0.172	−0.123	0.031
baPWV	−0.129	0.024	−0.104	0.049	−0.047	0.412	0.130	0.020

*Abbreviations*: *SVR* skeletal muscle mass to visceral fat area ratio, *ASM* appendicular skeletal muscle, *VFA* visceral fat area, *BMI* body mass index, *SBP* systolic blood pressure, *DBP* diastolic blood pressure, *TC* total cholesterol, *TG* triglyceride, *HDL-C* high-density lipoprotein cholesterol, *LDL-C* low-density lipoprotein cholesterol

### Comparison of the incidence of high baPWV in different tertiles of SVR

**Fig. 2:**
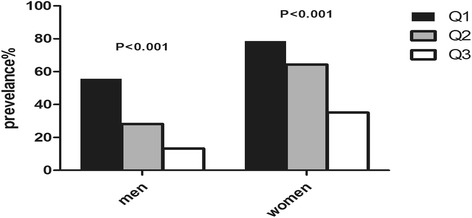
Prevalence of high baPWV across tertiles of SVR in Chinese type 2 diabetic patients. Patients were divided into tertiles based on admission SVR values. Among men, the prevalence of high baPWV in the Q1, Q2, Q3 levels was 56.1, 29.1, 17.9% respectively. Among women, the prevalence of high baPWV in the Q1, Q2, Q3 levels was 76.8, 63.2, 39.7%, respectively (*P* < 0.05)

The prevalence of high baPWV tended to decline with increasing levels of SVR (Fig. 2). Women had a higher prevalence of baPWV than men. Among men, the prevalence of high baPWV in the Q1, Q2, Q3 levels was 56.1, 29.1, 17.9% respectively. Among women, the prevalence of high baPWV in the Q1, Q2, Q3 levels was 76.8, 63.2, 39.7%, respectively (*P* < 0.05).

### Logistic regression analyses for baPWV

Logistic regression analyses to examine the relationship between the tertiles of SVR and baPWV are listed in Table 3. In an unadjusted model, the odds ratio for high baPWV was 8.18 (95% confidential interval, 3.78–17.70) in men and 6.78 in women (95% confidential interval, 2.93–15.69) in the lowest SVR tertile, compared to the highest SVR tertile. Although adjusting for age, duration of diabetes, duration of hypertension, BMI, waist circumference, SBP, DBP, HbA1c, smoking status, alcohol intake, FINS, uric acid, TC, TG, HDL-C, LDL-C attenuated the magnitude of the odds ratio, the association between SVR and high baPWV remained significant (odds ratio 4.33 in men and 4.66 in women, *P* < 0.01).

**Table 3:** multivariable-adjusted odd ratios for high baPWV accordingly to each tertile of SVR

	Univariate		Model1		Model2	
	OR	P	OR	P	OR	P
Men						
Q1	8.18(3.78–17.70)	0.00	3.30(1.40–7.80)	0.01	4.33(1.06–17.78)	0.04
Q2	2.58(1.17–5.68)	0.02	1.40(0.59–3.30)	0.44	1.25(0.32–4.92)	0.75
Q3	1(reference)	–	1	–	1	–
Women						
Q1	6.78(2.93–15.69)	0.00	3.63(1.45–9.10)	0.01	4.66(1.06–20.57)	0.04
Q2	3.33(1.54–7.20)	0.00	1.96(0.84–4.53)	0.12	3.60(1.00–12.91)	0.05
Q3	1	–	1	–	1	–

Model1: adjusted for age

Model2: further adjusted for duration diabetes, duration of hypertension, BMI, waist, SBP, DBP, HbA1c, smoker, alcohol, FINS, uric, TC, TG, HDL-C, LDL-C

*Abbreviations*: *SVR* skeletal muscle mass to visceral fat area ratio, *BMI* body mass index, *SBP* systolic blood pressure, *DBP* diastolic blood pressure, *FINS* fasting serum insulin, *TC* total cholesterol, *TG* triglyceride, *HDL-C* high-density lipoprotein cholesterol, *LDL-C* low-density lipoprotein cholesterol

### ROC curves of body composition area for high baPWV

**Fig. 3:**
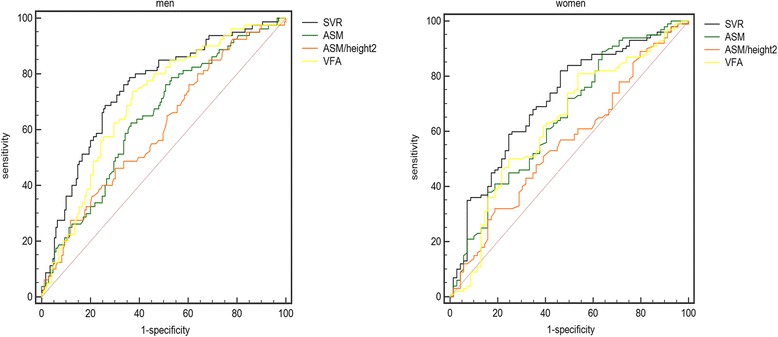
ROC curves of SVR, ASM, ASM/height2 and VFA for high baPWV. SVR had the highest area under the ROC curve, with statistical significance (0.747 in men and 0.710 in women)

To compare the predictability of a high baPWV, ROC curves for SVR, ASM, ASM/height^2^, and VSA were plotted (see in Fig. 3 and Table 4). SVR had the highest area under the ROC curve, with statistical significance (0.747 in men and 0.710 in women); the optimal cutoffs of SVR for detecting a high baPWV were 191.7 g/cm^2^ in men and 157.3 g/cm^2^ in women.

**Table 4:** ROC analysis between arterial stiffness versus SVR, ASM, ASM/height2 and VFA

	Cut-off	Sensitivity	Specificity	AUC	95%CI	P
Male						
SVR	191.7	0.74	0.687	0.747	0.688–0.800	0.000
ASM	21.52	0.633	0.625	0.643	0.580–0.702	0.000
ASM/height2	8.14	0.314	0.85	0.604	0.541–0.666	0.008
VFA	105.55	0.738	0.627	0.700	0.639–0.756	0.000
Female						
SVR	157.3	0.536	0.82	0.710	0.635–0.777	0.000
ASM	17.16	0.362	0.89	0.648	0.571–0.719	0.001
ASM/height2	5.95	0.826	0.31	0.558	0.480–0.634	0.196
VFA	97.15	0.802	0.457	0.630	0.553–0.703	0.008

*Abbreviations*: *SVR* skeletal muscle mass to visceral fat area ratio, *ASM* appendicular skeletal muscle, *VFA* visceral fat area

## Discussion

In this study, we found that baPWV increased with decreasing SVR levels. Furthermore, SVR was independently associated with arterial stiffness in Chinese patients with T2DM, even after adjusting for age, duration diabetes, duration of hypertension, BMI, waist circumference, SBP, DBP, HbA1c, smoking status, alcohol intake, FINS, uric acid level, TC, TG, HDL-C, LDL-C. Additionally, we found that SVR was a more powerful indicator of baPWV, than ASM, ASM/height^2^, and VFA.

Low skeletal muscle mass and high visceral fat, are known to be associated with atherosclerosis and cardiovascular diseases (CVDs) [2, 4]. In addition, some surveys showed that in western non-diabetic populations, SVR was negatively related to arterial stiffness [14]. However, this association has not been explored in Chinese T2DM patients, and it is uncertain whether SVR could predict atherosclerosis better than skeletal muscle mass or visceral fat. People with type 2 diabetes often have excess body fat mass and show accelerated muscle mass loss [15, 16], which is termed as sarcopenic obesity, and CVD in individuals with type 2 diabetes might be synergistically affected by a combination of high fat mass and low muscle mass. SVR, and not absolute amount of skeletal muscle mass or visceral fat, might be more appropriate to predict atherosclerosis in patients of diabetes mellitus. Fat mass should be considered in evaluating skeletal muscle mass in these subjects as reported previously [17]. While our study confirmed this hypothesis, as shown in Fig. 1, SVR had the largest area under the ROC curve compared with ASM, ASM/height^2^, and VSA, reaching diagnostic significance. Therefore, SVR has an advantage over conventional diagnostics in the screening of arterial stiffness in T2DM.

Ageing is associated with a decrease in skeletal muscle mass and an increase in body fat [18]. Similarly, in our study, a negative association between age and SVR was also observed in both men and women (*r* = − 0.304 in men and − 0.459 in women). Previous studies have demonstrated the relationship between SVR and CVD risk in individuals with no diabetes. According to the observation of Lim et al. on the Korean adult population, it has been found that among subjects with metabolic syndrome and arterial stiffness, the visceral fat to thigh muscle area ratio is increased dramatically [19]. The study of Kim et al. revealed a significantly association between the muscle mass/fat mass ratio with waist circumference, blood pressure, lipid profiles, and blood glucose levels [14]. In this study, as is shown in Table 2, we additionally showed that lower SVR is associated with CVD risk factors such as BMI (*r* = − 0.169, *P* = 0.003), DBP (*r* = − 0.132, *P* = 0.021), and waist circumference (*r* = − 0.119, *P* = 0.036) among subjects with T2DM after adjusting for age and gender. Given the multiple CVD risk factors associated with atherosclerosis, a lower SVR indicates the need for more aggressive efforts to manage atherosclerosis.

We found that both men and women with lower SVR had a higher prevalence of high baPWV than did those in the higher SVR group, and women had a higher prevalence than men. Previous studies have reported this finding. Newman et al. argued that women have less muscle mass and high visceral fat compared with men and they may be at a higher risk for sarcopenic obesity-related functional limitations and disability [17, 20]. In addition, most women in this study were postmenopausal, with a reduction and loss of muscle mass [21], and hence at a greater risk of developing atherosclerosis.

As an index of sarcopenic obesity, the reason why SVR may be associated with baPWV can be explained by several plausible mechanisms. To begin with, as the main site for insulin-mediated glucose disposal, low skeletal muscle might be correlated with insulin resistance [22]. In addition, there is a correlation between increased visceral fat and insulin resistance [23]. As a result, there might be a association between low SVR and insulin resistance. Next, a good correlation between both low muscle mass, fat mass and inflammatory cytokines was revealed by recent investigations [24]. As a result, pro-inflammatory status might be reflected by low SVR. What’s more, due to the connection between decreased muscle mass, increased visceral fat and low physical activity [25], it can be speculated that there was a correlation between low SVR and a sedentary life style. Thus, the pathophysiology of the important relationship between SVR and baPWV might be explained by insulin resistance, inflammation, and physical inactivity. In our study, logistic regression analysis using wide range of covariates showed that the odds ratio for high baPWV was 4.33 times higher in the lower SVR tertile compared to the higher SVR tertile in men, and 4.66 times in the women groups.

Considering that body compositions differ by ethnicity, with Asians having comparatively low muscle mass but high body fat, sarcopenic obesity poses a serious problem [26]. Consequently, further studies are required to evaluate the superiority of SVR, reflecting a condition resembling sarcopenic obesity, for predicting several cardiometabolic risk factors (such as dysglycemia, high blood pressure, dyslipidemia) in Asians, and to define the optimal cutoffs of SVR for cardio-metabolic risk factors in this population.

There are several limitations to this study. First, we used baPWV for the assessment of arterial stiffness. According to a previous study, carotid-femoral PWV is viewed as the “gold standard” for arterial stiffness evaluation [27]. Besides, the impact of baPWV on total cardiovascular events and mortality was revealed by a recent meta-analysis [28]. Consequently, we believe that the association between arterial stiffness defined by baPWV and SVR would have enough power to predict future cardiovascular events. Secondly, the dual bioelectrical impedance analyzer was used to measure VFA. Abdominal CT examination is also a gold standard for visceral adiposity assessment; however, in a previous report, an obvious correlation between VFA measured by CT and that measured by a dual bioelectrical impedance analyzer (*r* = 0.821, *P* < 0.001) was revealed [29]. Moreover, comparing to the abdominal CT scan, the dual bioelectrical impedance analyzer do have some advantages, for example, using dual bioelectrical impedance analyzer can avoid the risk of exposure to ionized radiation. Third, this study did not examine the impact of inflammatory marker and oxidative stress, and previous studies have confirmed that they are closely related to arterial stress [30, 31]. Fourth, in order to make definite conclusions, the sample size was far from enough. Finally, the cross-sectional as well as the longitudinal studies should be conducted to figure out the causal relationship between baPWV and SVR in T2DM.

## Conclusions

The present study showed for the first time that in both men and women, reduced SVR, an index of skeletal muscle mass corrected by visceral obesity, is independently associated with increased arterial stiffness in T2DM. SVR could be used as a more appropriate risk-assessment tool than ASM, ASM/height^2^, and VFA in clinical practice to identify patients with type 2 diabetes at high cardiovascular risk (the appropriate cutoff of SVR for high baPWV is 191.7 kg/cm^2^ in men and 157.3 kg/m^2^ in women).

## Abbreviations

ASM: Appendicular skeletal muscle
baPWV: brachial-ankle pulse wave velocity
BF: Total body fat percentage
BMI: Body mass index
DBP: Diastolic blood pressure
FINS: Fasting serum insulin
HbA1c: Glycated hemoglobin A1c
HDL-C: High-density lipoprotein cholesterol
HOMA-IR: Homeostatic model assessment of insulin resistance
LDL-C: Low-density lipoprotein cholesterol
SBP: Systolic blood pressure
SVR: Skeletal muscle mass to visceral fat area ratio
TC: Total cholesterol
TG: Triglyceride
VFA: Visceral fat area

## References

[1] Laakso M, Lehto S (1998). Epidemiology of risk factors for cardiovascular disease in diabetes and impaired glucose tolerance. Atherosclerosis.

[2] Kim Y, Han BD, Han K, Shin KE, Lee H, Kim TR, Cho KH, Kim DH, Kim YH, Kim H (2015). Optimal cutoffs for low skeletal muscle mass related to cardiovascular risk in adults: the Korea National Health and nutrition examination survey 2009-2010. Endocrine.

[3] Terada T, Boule NG, Forhan M, Prado CM, Kenny GP, Prud'homme D, Ito E, Sigal RJ (2017). Cardiometabolic risk factors in type 2 diabetes with high fat and low muscle mass: at baseline and in response to exercise. Obesity.

[4] Dahlen EM, Tengblad A, Lanne T, Clinchy B, Ernerudh J, Nystrom FH, Ostgren CJ (2014). Abdominal obesity and low-grade systemic inflammation as markers of subclinical organ damage in type 2 diabetes. Diabetes Metab.

[5] Tian S, Xu Y (2016). Association of sarcopenic obesity with the risk of all-cause mortality: a meta-analysis of prospective cohort studies. Geriatr Gerontol Int.

[6] Kohara K (2014). Sarcopenic obesity in aging population: current status and future directions for research. Endocrine.

[7] Wykretowicz A, Gerstenberger P, Guzik P, Milewska A, Krauze T, Adamska K, Rutkowska A, Wysocki H (2009). Arterial stiffness in relation to subclinical atherosclerosis. Eur J of Clin Invest.

[8] Palombo C, Kozakova M (2016). Arterial stiffness, atherosclerosis and cardiovascular risk: pathophysiologic mechanisms and emerging clinical indications. Vasc Pharmacol.

[9] Mahe G, Kaladji A, Le Faucheur A, Jaquinandi V (2016). Internal iliac artery disease management: still absent in the update to TASC II (inter-society consensus for the Management of Peripheral Arterial Disease). J Endovasc Ther.

[10] Ida M, Hirata M, Odori S (2013). Early changes of abdominal adiposity detected with weekly dual bioelectrical impedance analysis during calorie restriction. Obesity.

[11] Heymsfield SB, Smith R, Aulet M, Bensen B, Lichtman S, Wang J, Pierson RN (1990). Appendicular skeletal muscle mass: measurement by dual-photon absorptiometry. Am J Clin Nutr.

[12] Cruz-Jentoft AJ, Baeyens JP, Bauer JM, Boirie Y, Cederholm T, Landi F, Martin FC, Michel JP, Rolland Y, Schneider SM (2010). Sarcopenia: European consensus on definition and diagnosis: report of the European working group on sarcopenia in older people. Age Ageing.

[13] Tomiyama H, Koji Y, Yambe M, Shiina K, Motobe K, Yamada J, Shido N, Tanaka N, Chikamori T, Yamashina A (2005). Brachial -- ankle pulse wave velocity is a simple and independent predictor of prognosis in patients with acute coronary syndrome. Circ J.

[14] Kim TN, Park MS, Lim KI, Yang SJ, Yoo HJ, Kang HJ, Song W, Seo JA, Kim SG, Kim NH (2011). Skeletal muscle mass to visceral fat area ratio is associated with metabolic syndrome and arterial stiffness: the Korean Sarcopenic obesity study (KSOS). Diabetes Res Clin Pract.

[15] Park SW, Goodpaster BH, Strotmeyer ES, Kuller LH, Broudeau R, Kammerer C, de Rekeneire N, Harris TB, Schwartz AV, Tylavsky FA (2007). Accelerated loss of skeletal muscle strength in older adults with type 2 diabetes: the health, aging, and body composition study. Diabetes Care.

[16] Volpato S, Bianchi L, Lauretani F, Lauretani F, Bandinelli S, Guralnik JM, Zuliani G, Ferrucci L (2012). Role of muscle mass and muscle quality in the association between diabetes and gait speed. Diabetes Care.

[17] Newman AB, Kupelian V, Visser M, Simonsick E, Goodpaster B, Nevitt M, Kritchevsky SB, Tylavsky FA, Rubin SM, Harris TB (2003). Sarcopenia: alternative definitions and associations with lower extremity function. J Am Geriatr Soc.

[18] Roubenoff R (2000). Sarcopenic obesity: does muscle loss cause fat gain? Lessons from rheumatoid arthritis and osteoarthritis. Ann N Y Acad Sci.

[19] Lim KI, Yang SJ, Kim TN, Yoo HJ, Kang HJ, Song W, Baik SH, Choi DS, Choi KM (2010). The association between the ratio of visceral fat to thigh muscle area and metabolic syndrome: the Korean Sarcopenic obesity study (KSOS). Clin Endocrinol.

[20] Dos Santos EP, Gadelha AB, Safons MP, Nobrega OT, Oliveira RJ, Lima RM (2014). Sarcopenia and sarcopenic obesity classifications and cardiometabolic risks in older women. Arch Gerontol Geriatr.

[21] Messier V, Rabasa-Lhoret R, Barbat-Artigas S, Elisha B, Karelis AD, Aubertin-Leheudre M (2011). Menopause and sarcopenia: a potential role for sex hormones. Maturitas.

[22] de Matos M, de Ottone V, Duarte T, da Sampaio P, Costa K, Fonseca C, Neves M, Schneider S, Moseley P, Coimbra C (2013). Exercise reduces cellular stress related to skeletal muscle insulin resistance. Cell Stress Chaperones.

[23] Medina-Urrutia A, Posadas-Romero C, Posadas-Sanchez R, Jorge-Galarza E, Villarreal-Molina T, Gonzalez-Salazar Mdel C, Cardoso-Saldana G, Vargas-Alarcon G, Torres-Tamayo M, Juarez-Rojas JG. Role of adiponectin and free fatty acids on the association between abdominal visceral fat and insulin resistance. Cardiovasc Diabetol. 2015; 10.1186/s12933-015-0184-5. 10.1186/s12933-015-0184-5 PMC4332423 25849597

[24] Cesari M, Kritchevsky SB, Baumgartner RN, Atkinson HH, Penninx BW, Lenchik L, Palla SL, Ambrosius WT, Tracy RP, Pahor M (2005). Sarcopenia, obesity, and inflammation--results from the trial of angiotensin converting enzyme inhibition and novel cardiovascular risk factors study. Am J Clin Nutr.

[25] Ryall JG, Schertzer JD, Lynch GS (2008). Cellular and molecular mechanisms underlying age-related skeletal muscle wasting and weakness. Biogerontology.

[26] Rush EC, Goedecke JH, Jennings C, Micklesfield L, Dugas L, Lambert EV, Plank LD (2007). BMI, fat and muscle differences in urban women of five ethnicities from two countries. Int J Obes.

[27] Mattace-Raso F, Hofman A, Verwoert GC (2010). Determinants of pulse wave velocity in healthy people and in the presence of cardiovascular risk factors: ‘establishing normal and reference values’. Eur Heart J.

[28] Vlachopoulos C, Aznaouridis K, Terentes-Printzios D, Ioakeimidis N, Stefanadis C (2012). Prediction of cardiovascular events and all-cause mortality with brachial-ankle elasticity index: a systematic review and meta-analysis. Hypertension.

[29] Yamashina A, Tomiyama H, Takeda K, Tsuda H, Arai T, Hirose K, Koji Y, Hori S, Yamamoto Y (2002). Validity, reproducibility, and clinical significance of noninvasive brachial-ankle pulse wave velocity measurement. Hypertension Res.

[30] Mozos I, Malainer C, Horbanczuk J, Gug C, Stoian D, Luca CT, Atanasov AG. Inflammatory markers for arterial stiffness in cardiovascular diseases. Front Immunol. 2017; 10.3389/fimmu.2017.01058. 10.3389/fimmu.2017.01058 PMC5583158 28912780

[31] Mozos I, Luca CT (2017). Crosstalk between oxidative and Nitrosative stress and arterial stiffness. Curr Vasc Pharmacol.

